# Dimethyl Fumarate Mediates Sustained Vascular Smooth Muscle Cell Remodeling in a Mouse Model of Cerebral Aneurysm

**DOI:** 10.3390/antiox13070773

**Published:** 2024-06-27

**Authors:** Alejandra N. Martinez, Giovane G. Tortelote, Crissey L. Pascale, Uduak-Obong I. Ekanem, Ana Paula de O. Leite, Isabella G. McCormack, Aaron S. Dumont

**Affiliations:** 1 Department of Neurosurgery, The Tulane Center for Clinical Neurosciences, Tulane University School of Medicine, 1430 Tulane Avenue, New Orleans, LA 70012, USAadumont2@tulane.edu (A.S.D.); 2 Department of Pediatrics, Tulane University School of Medicine, New Orleans, LA 70112, USA; gtortelote@tulane.edu; 3 Department of Pharmacology, The Tulane Center for Sex-Based Biology and Medicine, Tulane University School of Medicine, New Orleans, LA 70112, USA

**Keywords:** cerebral aneurysms, dimethyl fumarate, vascular smooth muscle cell, mitochondrial function, glutathione redox system

## Abstract

Cerebral aneurysms (CA) are a type of vascular disease that causes significant morbidity and mortality with rupture. Dysfunction of the vascular smooth muscle cells (VSMCs) from circle of Willis (CoW) vessels mediates CA formation, as they are the major cell type of the arterial wall and play a role in maintaining vessel integrity. Dimethyl fumarate (DMF), a first-line oral treatment for relapsing-remitting multiple sclerosis, has been shown to inhibit VSMC proliferation and reduce CA formation in a mouse model. Potential unwanted side effects of DMF on VSMC function have not been investigated yet. The present study characterizes the impact of DMF on VSMC using single-cell RNA-sequencing (scRNA-seq) in CoW vessels following CA induction and further explores its role in mitochondrial function using in vitro VSMC cultures. Two weeks of DMF treatment following CA induction impaired the transcription of the glutathione redox system and downregulated mitochondrial respiration genes in VSMCs. In vitro, DMF treatment increased lactate formation and enhanced the mitochondrial production of reactive oxygen species (ROS). These effects rendered VSMCs vulnerable to oxidative stress and led to mitochondrial dysfunction and enhancement of apoptosis. Taken together, our data support the concept that the DMF-mediated antiproliferative effect on VSMCs is linked to disturbed antioxidative functions resulting in altered mitochondrial metabolism. This negative impact of DMF treatment on VSMCs may be linked to preexisting alterations of cerebrovascular function due to renal hypertension. Therefore, before severe adverse effects emerge, it would be clinically relevant to develop indices or biomarkers linked to this disturbed antioxidative function to monitor patients undergoing DMF treatment.

## 1. Introduction

Cerebral aneurysms (CA) are a type of vascular disease that causes significant morbidity and mortality with rupture. Dysfunction of the vascular smooth muscle cells (VSMCs) from circle of Willis (CoW) vessels mediates CA formation, as they are the major cell type of the arterial wall and play a role in maintaining vessel integrity via the production of extracellular matrix components [1]. Traditionally, VSMC phenotypes are classified as proliferative synthetic or quiescent contractile, which represent two ends of a spectrum [2]. However, these cells are not terminally differentiated and present a remarkable degree of plasticity. With the development of single-cell RNA sequencing (scRNA-seq), emerging studies reveal extensive heterogeneity among VSMCs in healthy and diseased vessels [3,4,5,6,7]. Currently, no consensus exists regarding the number of potential VSMC phenotypes, but a recent review based on scRNA-seq literature classified them into the following six types: contractile, mesenchymal-like, fibroblast-like, macrophage-like, osteogenic-like, and adipocyte-like [6].

Dimethyl fumarate (DMF), a cell-permeable analog of fumarate and a first-line oral treatment for relapsing-remitting multiple sclerosis, has been shown to inhibit VSMC proliferation [8,9]. Previously, using a rodent elastase model of CA, we demonstrated that DMF significantly decreased the incidence of aneurysm formation [8]. This beneficial effect of DMF resulted in less oxidative stress, inflammation, and fibrosis in the cerebrovasculature of CA-induced mice. Using VSMC in vitro cultures, we also demonstrated that DMF protected the cells from TNF-α-induced inflammation and inhibited its pro-proliferative action by increasing apoptosis. DMF’s primary mechanism of action involves activation of the transcription factor nuclear factor erythroid 2-related factor 2 (Nrf2), although additional Nrf2-independent pathways have been described [10,11,12]. Several studies have highlighted the multifaceted utility of DMF; however, there is still limited understanding of the role of Nrf2 in governing VSMC phenotypes in physiological and pathological conditions.

The biological consequence of Nrf2 activation can be either protective or detrimental, and this contradiction relies on model discrepancies, as well as whether Nrf2 is activated in an acute or chronic manner. This dual role of Nrf2 on VSMCs can contribute to either vascular repair or disease [13]. Most studies have focused on the protective properties of Nrf2, mainly as the key transcription factor in antioxidant defense. However, multiple terminated clinical trials have shown a negative side to exogenous Nrf2 activation with regard to cardiac pathologies, and animal-based studies have demonstrated cardiomyocyte hypertrophy and heart failure after chronic Nrf2 upregulation [13,14,15,16]. Here, we performed scRNA-seq in CoW vessels to study the impact of Nrf2 activation by DMF in VSMCs following CA induction and further explored its role in mitochondrial function using in vitro VSMC cultures. We found that two weeks of DMF treatment impairs the glutathione redox system in VSMCs and causes changes in mitochondrial respiration. This effect renders VSMCs vulnerable to oxidative stress and leads to mitochondrial dysfunction and enhancement of apoptosis.

## 2. Materials and Methods

For a comprehensive description of the methods, see Supplemental Material.

### 2.1. Mouse Model of Elastase-Induced CA and DMF Treatment

Animal research was performed in accordance with the Tulane University IACUC (Protocol ID: 1607). Eight-week-old male C57BL/6 mice were obtained (Jackson Laboratories, Bar Harbor, ME, USA). To induce aneurysms, we used a well-established model that involves pharmacological hypertension and stereotactic injection of elastase [17]. Briefly, male mice underwent a unilateral nephrectomy followed by implantation of a deoxycorticosterone acetate pellet in the subcutaneous tissue 1 week later. The mice were given 1% NaCl in their drinking water, and 17.5 mU of elastase was injected into the basal cistern.

Male mice were randomly assigned to be treated with either DMF (Sigma Aldrich, St. Louis, MI, USA, 242,926) at 100 mg/kg/day orally or vehicle, carboxymethylcellulose (CMC) (Sigma Aldrich, C9481), from the time of CA induction until sacrifice 2 weeks later. CMC was used as a thickening agent to form a colloidal mixture with DMF, which was administered by oral gavage at a volume of 0.2 mL. There was an additional cohort of sham-operated controls. Female mice were not used because previous work in our lab has shown no significant differences in aneurysm prevalence between sexes at this age. Moreover, published research articles have shown sex differences only in ovariectomized mice [18]. At the conclusion of the study, the mice were sacrificed, and the brains were placed under a microscope to note whether aneurysms had formed. Researchers were blinded to treatment groups. The CoW vessels were then harvested for scRNA-seq.

### 2.2. CoW Dissociation and Single-Cell Preparation

Preparation of the single-cell suspension was performed following a previously described protocol [7]. Cerebral blood vessels from the CoW were harvested, and the connective and arachnoid tissue removed. The CoW pooled from 5 mice in each group was digested with an enzyme solution. The cell suspension was strained, washed, and resuspended in opti-MEM with 10% FBS.

### 2.3. Single-Cell RNA-Sequencing Data Analysis

The cell suspensions underwent scRNA-seq using standard 10× Chromium Single Cell Chemistry V3. Raw sequencing data were processed using Cell Ranger version 6.0.1 to produce gene-level counts for each cell in each sample. The CellRanger mkfastq command was used to generate Fastq files. Data were mapped to a prebuilt mouse reference genome. All subsequent analysis was performed using R (Version 4.4.1). See Supplementary Methods for a detailed description of the scRNA-seq pipeline.

The scRNAseq data generated in this study have been deposited in the Gene Expression Omnibus (GEO) database under the accession codes GSE193533 and GSE268079.

### 2.4. Vascular Smooth Muscle Cell Culture and DMF Treatment

C57BL/6 mouse primary cerebral VSMCs (cat. # C57-6085, Cell Biologics, Chicago, IL, USA) were cultured in DMEM (low glucose) supplemented with 10% (*v*/*v*) FBS and antibiotics (100 U/mL penicillin, 100 mg/mL streptomycin) at 37 °C in a humidified, 5% CO_2_ incubator. Cells between the third and sixth passages were used in all experiments. VSMCs were plated in 12-well plates at 1 × 10^5^ cells/well and maintained in media containing DMEM/F12 and 10% FBS. They were subsequently treated with TNFα (50 ng/mL) and/or DMF (10 μM, 25 μM and 50 μM; Sigma-Aldrich, T5944) in culture medium containing ≤0.001% DMSO for 4 or 24 h. The dose of TNFα was chosen based on prior publications from our group [8].

To better mimic the CA mouse model and understand the impact of DMF treatment in vitro, we did not use quiescent cells. Instead, cells were kept in media containing 10% FBS, as this reflects the proliferative potential state linked to hypertension (a step required in the CA mouse model).

### 2.5. Mitochondrial Stress Assay

Mitochondrial stress assay (cat. #103020-100, Agilent, Santa Clara, CA, USA) was performed using an XF24 Extracellular Flux Analyzer (Agilent) following the workflow provided by the manufacturer’s instructions. Briefly, for oxygen consumption rate (OCR) and extracellular acidification rate (ECAR) measurements, VSMCs were seeded in quadruplets on XF24 microplates at a density of 1 × 10^4^ cells per well in assay medium (XF DMEM medium pH 7.4 supplemented with 10 mM glucose, 2 mM glutamine, 1 mM pyruvate), followed by incubation at 37 °C in a non-CO_2_ incubator for 60 min. Three baseline measurements were recorded before the injection of the following compounds: Oligomycin in port A (56 μL) at 1.0 μM, FCCP in port B (62 μL) at 1.0 μM, and Rotenone/Antimycin in port C (69 μL) at 0.5 μM. Data analysis was performed using Cell Mito Stress Test Report Generators software (Wave 2.6.3).

### 2.6. ATP Rate Assay

For the ATP production rate assay, an Agilent Seahorse XF Real-Time ATP Rate Assay Kit (cat. #103592-100) was used. VSMCs were plated as described above, and ATP measurements were recorded, followed by Oligomycin injection in port A (56 μL) at 1.5 μM final concentration and Rotenone/Antimycin injection in port B (62 μL) at 0.5 μM final concentration, as indicated in the manufacture’s instructions. Data analysis was performed using Report Generators software for Real-Time ATP Rate Assay.

### 2.7. L-Lactate Release Assay

VSMC L-lactate release from the medium was collected at 4 and 24 h after treatment using the Lactate-Glo™ Assay (cat. #J5021, Promega, MD, USA) according to the manufacturer’s instructions. Luminescence was measured using a microplate reader (SpectraMax M5, Molecular Devices, San Jose, CA, USA).

### 2.8. Glutathione Determination

Total glutathione (GSH) and oxidized glutathione (GSSG) were determined using a Glutathione Colorimetric Detection Kit (cat. # EIAGSHC, Thermo Fisher Scientific, Waltham, MA, USA). Briefly, cells were cultured in 12-well plate at a density of 3 × 10^5^ cells/well and incubated with TNFα and DMF for 4 or 24 h. Cells were then washed with ice-cold PBS and resuspended in 200 μL of ice-cold 5% aqueous 5-sulfo-salicylic acid dehydrate (SSA), followed by sonication. Lysed cells were centrifuged at 14,800× *g* at 4 °C for 15 min, and 2-vinylpyridine (2VP) was used to block GSH and also measure GSSG content. The assay was conducted by following the kit instructions. Absorbance at 405 nm was measured using a microplate reader (SpectraMax M5, Molecular Devices).

### 2.9. MitoTracker Mitochondrion-Selective Probes

Primary VSMC were cultured in 12-well plates at a density of 3 × 10^5^ cells/well and treated as described above. After 4 or 24 h of incubation, cells were trypsinized and labeled with 150 nM green-fluorescing MitoTracker Green (cat. #M7514, Thermo Fisher Scientific) to measure mitochondrial content, and 200 nM MitoTracker Red CMXRos (cat. #M7512) to measure mitochondrial membrane potential according to a published protocol [19]. MitoTracker probes were incubated for 30 min at 37 °C, and then, cells were washed twice with Live Cell Imaging Solution (cat. #A14291DJ, Thermo Fisher Scientific) and analyzed on a flow cytometer.

### 2.10. Annexin V-FITC Staining for Apoptosis Detection

The rate of VSMC cell apoptosis after DMF treatment was evaluated using Annexin V-FITC (cat. #A13199, Thermo Fisher Scientific). Following treatment with different concentrations of TNFα and DMF, the cells were harvested, washed with PBS and resuspended in 100 µL binding buffer. Then, the cells were incubated in the dark with Annexin V for 10 min and analyzed using flow cytometry.

### 2.11. Statistical Analysis

In experiments other than scRNA-seq, analysis was performed using GraphPad Prism 10.0 Software where each treatment group was compared to the control group. For cell culture assays, two biological replicates with three technical replicates were used. Normality was examined using Shapiro-Wilk test and statistical tests were performed using continuous normal distributed data. Individual statistical tests for each dataset are indicated in the figure legends. Two-way (Figures 6–8) or one-way (Figure 10) ANOVA with Dunnett’s post hoc against the control group was used. *p* < 0.05 was taken as statistically significant.

## 3. Results

### 3.1. VSMC Fate and States

**Figure 1:**
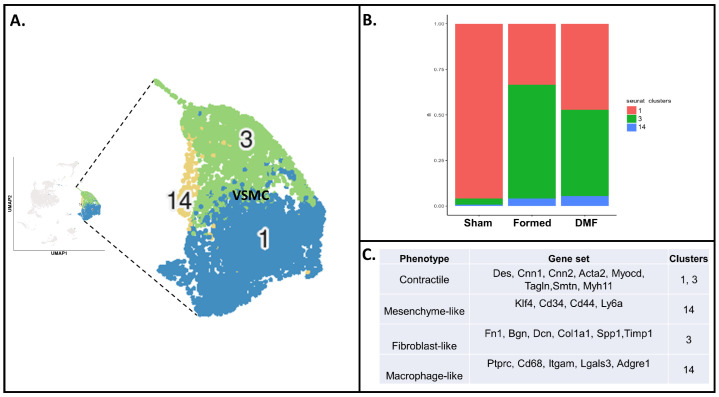
Identification of VSMC clusters present in the mouse circle of Willis (CoW) by single-cell RNA sequencing (scRNAseq). (**A**) UMAP plot of aggregate CoW cells with colors denoting different VSMC clusters. (**B**) VSMC population percentages across conditions. (**C**) Clusters and phenotypes of correspondence.

We used single-cell transcriptomics to gain insight into the VSMC fate and states after two weeks of CA induction and DMF treatment. To delineate VSMC heterogeneity, we first subsetted the cells that showed consistently high expression of the VSMC markers *Myh11*, *Acta2* and *Tagln* [7] and obtained a total of three distinct populations (clusters 1, 3 and 14; Figure 1A). Subsequently, the cellular counts were balanced to make them equal and comparable between the three treatments: sham, animals that formed CA after induction (formed) and animals that were treated with DMF after CA induction (DMF). A total of 528 cells per treatment were used to distinguish and classify the three VSMC clusters. VSMCs from sham animals corresponded mainly to cluster 1 (95.8%). In the “formed” and “DMF” groups, cluster 1 corresponded to 33.5% and 47.2% of VSMCs, respectively. Cluster 3 was more abundant in the formed group (62.3%) compared to the DMF group (47.3%), and cluster 14 had a similar proportion in both groups (4.2 and 5.5%, respectively) (Figure 1B).

**Figure 2:**
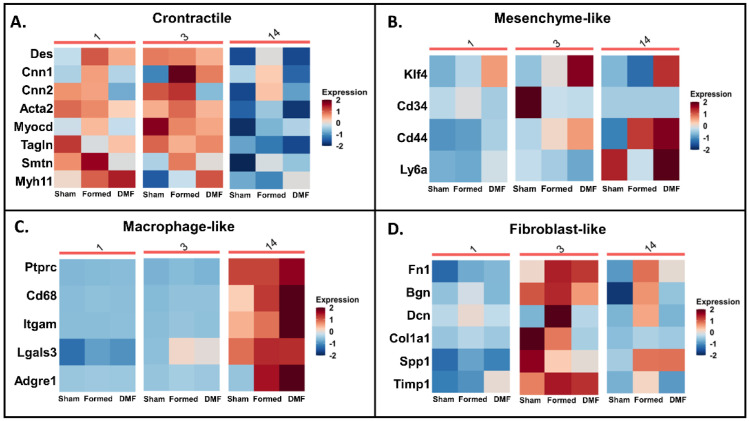
VSMC phenotypic diversity. Heatmap showing relative gene expression levels (columns) of the indicated metabolic pathways in VSMC subpopulations and across conditions. (**A**) Contractile, (**B**) Mesenchyme-like, (**C**) Macrophage-like, (**D**) Fibroblast-like.

Next, to address molecular and functional characteristics of different populations of VSMCs under physiological (sham) and pathological (CA) conditions, and to determine the effect of DMF treatment, we categorized the clusters based on previously established markers [6]. Furthermore, the three distinct VSMC clusters were classified based on expression of specific markers involved in phenotype modulation (Figure 1C and Figure 2). As expected, healthy VSMCs (sham) showed higher expression of contractile markers (cluster 1 and 3; Figure 2A). Sham also presented with an increased expression of fibroblast and macrophage-like markers in clusters 3 and 14, respectively (Figure 2C,D), although these represented only 4.2% of the total cells from this group. When CA was induced (formed), VSMCs exhibited an even more fibroblast-like phenotype in clusters 3 and 14 (Figure 2D) and a more macrophage-like phenotype in cluster 14 (Figure 2C). DMF treatment had a similar effect, with cluster 14 also expressing mesenchymal-like markers (Figure 2B). The transcription factor Krüppel-like factor 4 (Klf4) is known to play a pivotal role in the initial dedifferentiation of VSMCs to the mesenchymal-like phenotype, enabling further cellular changes towards other phenotypes [6,20,21]. Klf4 is also an important mediator of growth arrest following DNA damage [22]. Accordingly, DMF treatment significantly increased Klf4 expression in cells from clusters 3 and 14 (Figure S1), suggesting that it inhibits proliferation and pushes these cells into a de-differentiated self-renewing state. To confirm this, we quantified the thickening of the VSMC layer in CoW by quantifying SMA-α and confirmed that DMF treatment decreased SMA-α deposition (Figure S2), indicating proliferation inhibition.

**Figure 3:**
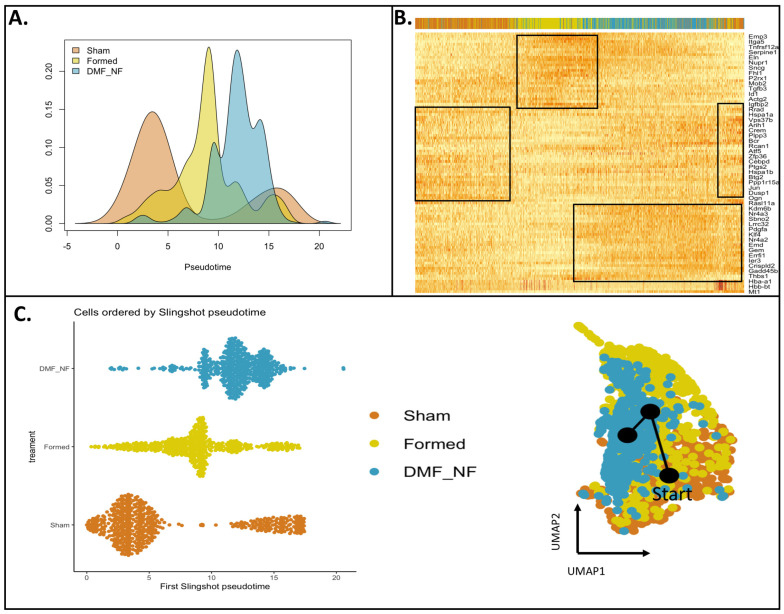
Pseudotemporal trajectory inference across VSMC conditions. A subset containing the same number of VSMCs per group was used as input for pseudotime and trajectory analysis with Slingshot R-package. (**A**) Differential progression, (**B**) Heatmap showing gene expression changes over pseudotime in all three treatment groups, (**C**) Cell ordering over pseudotime progression showing differences in ontogeny states induced by treatments (**left panel**). Inference of the global lineage structure and pseudotime using Slingshot on clustered data points (**right panel**).

To investigate how CA induction and treatment impacted the normal ontogeny of VSMCs, we performed trajectory inference using the Slingshot algorithm. Analysis showed that DMF treatment led to a distinct injury response when compared to the “formed” group. Using “sham” as the root of the trajectory, we found that “formed” shifted right from sham while “DMF” located to the end (far right) of the trajectory (Figure 3A,C). As expected, most cells from the “Sham” group had a lower pseudotime score characteristic of an undisturbed cell population. The shift and higher scores observed in the “Formed” and “DMF” groups indicate these cells were pushed to a more advanced state (Figure 3A,C). Next, we used pseudotime analysis to infer gene expression changes along the reconstructed trajectories. Genes showing significant changes (p-adj < 0.05, log(fold change) > |0.5|) were identified using generalized additive models (GAMs) and organized into gene clades based on Pearson correlation (Figure 3B and Figure S3). Similarities as well as some overlap were found along the trajectories of all three groups (Figure 3C and Figure S3). Genes associated with transcription regulation, cell cycle and transport (e.g., Btg2, Dusp1 and Zfp36) were specifically induced in the sham group, indicating regulation of genome stability. CA induction revealed enrichment of genes associated with blood vessel remodeling (e.g., Actg2, Eln, Itga5), while DMF treatment led to enrichment of genes related to stress response and transcription regulation (e.g., Hspa1a, Hspa1b, Klf4 and Atf5) (Figure S3).

**Figure 4:**
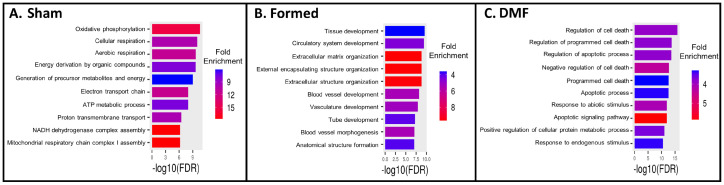
Gene ontology enrichment analysis of biological processes. Analysis of the top 100 DEGs from VSMCs across conditions using ShinnyGO 0.77 (**A**) Sham-operated group, (**B**) Formed group, (**C**) DMF group.

To evaluate the relevance of proliferation-associated, injury-induced and vascular repair genes in CA, we assessed the top100 DEGs in VSMCs from the three clusters combined (1, 3 and 14; Table S1). As expected, GO terms from the sham-operated group were associated with metabolic homeostasis (Figure 4A), reiterating that VSMCs mainly rely on oxygen-consuming mitochondrial oxidative phosphorylation (OXPHOS). OXPHOS ensures high efficiency of ATP production required for VSMC contractile function to maintain proper vascular tone, even though a substantial proportion of ATP still originates from glycolysis [23,24]. In the aneurysm “formed” group, GO terms displayed evidence of phenotypic shifting, as they related to extracellular matrix modification and vascular development (Figure 4B). Surprisingly, in the DMF treatment group, genes related to cell death were upregulated (Figure 4C), suggesting vascular toxicity. Discrepancies regarding the cytoprotective or cytotoxic effect of DMF in different cell types have been described and are thought to be, in part, dose-dependent [25]. Given these findings, we then proceeded to evaluate the functional consequences of DMF in VSMCs in vivo (using scRNA-seq pipeline) as well as in vitro.

### 3.2. DMF Treatment Leads to VSMC Respiratory Dysfunction

DMF could be expected to produce cardiac hypertrophy, as exogenous upregulation of Nrf2 beyond the control of repressive/degradation machinery may be deleterious [14]. scRNA-seq analysis showed that two weeks of DMF treatment in CA-induced mice upregulated Nrf2 and its main downstream targets in VSMCs (Figure S4). Persistent activation of the Nrf2 pathway can be attributed to nuclear-cytoplasmic loss of Keap1, a repressor protein that facilitates ubiquitin-proteasome degradation of Nrf2 in a p62-dependent manner [26]. Indeed, transcriptional levels of Keap1 were decreased in DMF-treated mice, while p62 (Sqstm1) was increased (Figure S4).

**Figure 5:**
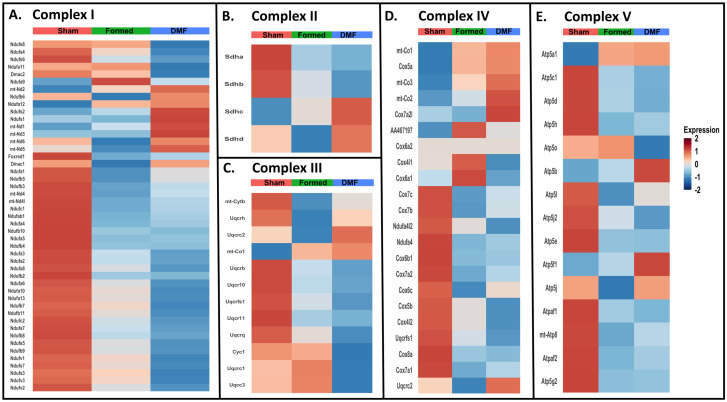
Mitochondrial OXPHOS complex module gene expression in VSMCs across conditions. Heatmaps for specific mitochondrial complex genes. (**A**) Complex I, (**B**) Complex II, (**C**) Complex III, (**D**) Complex IV, (**E**) Complex V.

We next investigated the interplay between DMF and the bioenergetic features of VSMCs using scRNA-seq data. Analysis revealed that aneurysm induction leads to downregulation of OXPHOS genes, across all five electron transport chain complexes, and that DMF treatment was not able to rescue mitochondrial gene expression (Figure 5A–E). Interestingly, mtDNA-encoded gene expression in the electron transport subunits following DMF treatment was increased (Figure S5), suggesting a compensatory response mechanism for the decline in mitochondrial function, which may actually be connected to enhanced ROS generation.

**Figure 6:**
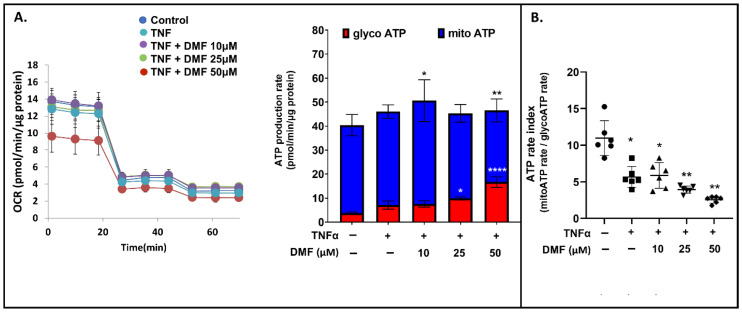
Seahorse XF real-time ATP rate analysis of VSMCs. (**A**) Left panel: Representative oxygen consumption rate (OCR) profile; Right panel: Mitochondrial and glycolytic ATP production rates in VSMCs treated with TNFα ± DMF. (**B**) XF ATP rate index calculated from data in panel A. All Seahorse data shown are compiled from three independent experiments using four technical replicates per experiment per condition and normalized by protein content (two-way ANOVA with Dunnett’s post hoc against the control group: **** *p* < 0.0001; ** *p* < 0.01; * *p* < 0.05).

The mechanism of action of DMF is complex and likely involves both antioxidant and oxidant effects. In MS patients, DMF was found to increase the oxidative environment in peripheral blood, distinguishing patients with beneficial treatment response from non-responders [27]. In general, if ROS levels exceed a certain threshold, they will impair OXPHOS complexes, further impacting mitochondrial respiration. Considering the above, we tested the capacity of DMF to modulate global energetic metabolism by measuring ATP production from glycolysis and mitochondria in cultured VSMCs treated with TNFα and DMF using Seahorse XF24 analyzer. Our data showed that the higher concentration of DMF (50 μM) significantly increased glycolysis-derived ATP, while it decreased mito-derived ATP (Figure 6A). Conversely, the lower concentration of DMF (10 μM) significantly increased mito-derived ATP, while glycolysis-derived ATP remained unchanged (Figure 6A). We then calculated the ATP rate index, which is the mitochondrial ATP production rate divided by the glycolytic ATP production rate at a given time point, and found a significant reduction when VSMCs were treated with DMF, indicating a shift toward glycolytic metabolism (Figure 6B). To investigate if the respiratory deficit induced by DMF was Nrf2-dependent, we treated VSMCs with ML385 (a pharmacological Nrf2 inhibitor). ML385 did not abrogate DMF-mediated effects on mito-derived ATP, but it resulted in reduced glycolytic dependency (Figure S6). This result highlights that a higher proportion of ATP generation is derived from glycolysis than mitochondrial respiration when cells are treated with DMF, supporting that DMF induces mitochondrial dysfunction in an Nrf2-dependent manner.

**Figure 7:**
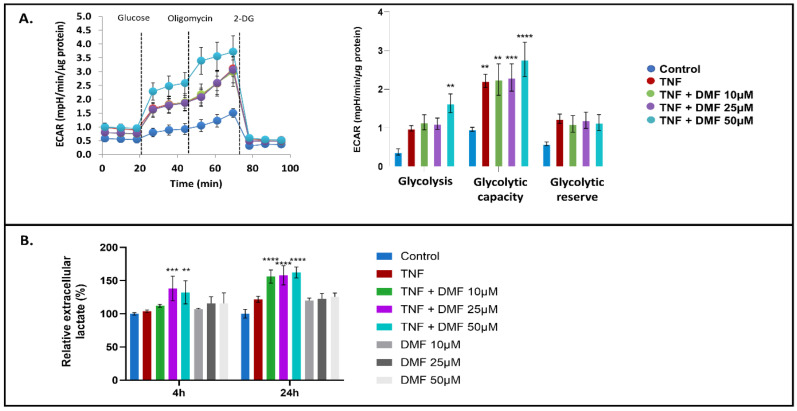
Seahorse XF real-time glycolysis stress test and extracellular lactate levels of VSMCs. (**A**) (**left panel**): Representative extracellular acidification rate (ECAR) profile (**left panel**); (**right panel**): Glycolytic bioenergetic parameters: basal glycolysis, glycolytic capacity and glycolytic reserve measured in VSMCs treated with TNFα ± DMF. (**B**) Relative levels of lactate in the culture supernatant of VSMCs treated for 4 and 24 h with TNFα ± DMF or DMF alone. All data shown are compiled from three independent experiments using four technical replicates per experiment per condition and normalized by protein content (two-way ANOVA with Dunnett’s post hoc against the control group: **** *p* < 0.0001; *** *p* < 0.001; ** *p* < 0.01).

Furthermore, the seahorse glycolysis stress test confirmed enhanced glycolysis when the higher concentration of DMF was used in the presence of TNFα (Figure 7A) but not in cells treated with DMF alone (Figure S7A). We also observed that DMF treatment significantly increased glycolytic capacity (Figure 7A). Since the glycolytic capacity is a measure of the maximum rate of conversion of glucose to pyruvate or lactate that can be achieved acutely by a cell, we next measured the release of extracellular lactate using a bioluminescent assay. Our results showed that DMF and TNFα cotreatment significantly increased lactate release (Figure 7B), but ML385 abolished this effect (Figure S7). These data indicate that DMF has a crucial impact on both mitochondrial respiratory activity and lactate accumulation in VSMCs, suggesting DMF increases ROS production in an Nrf2-dependent fashion.

### 3.3. Increased Cellular Respiratory Dysfunction by DMF Increases ROS Accumulation and Sensitizes VSMCs to Apoptosis

**Figure 8:**
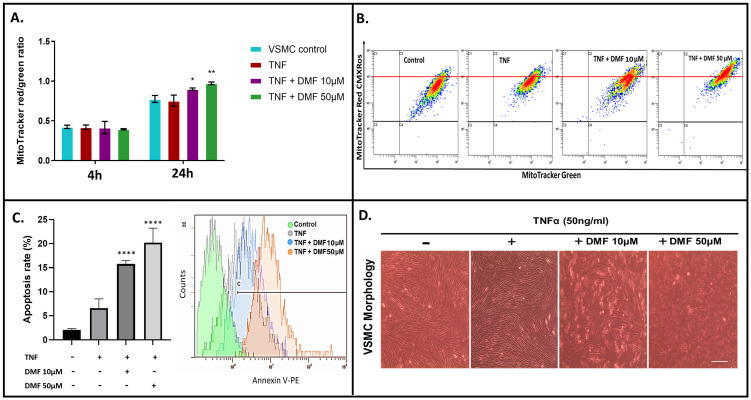
Mitochondrial dysfunction and cellular apoptosis. (**A**) VSMCs treated with TNFα ± DMF for 4 h were stained with MitoTracker Red CM-H2XRos and MitoTracker Green, and the fluorescence intensities were quantified using flow cytometry. The red:green ratio was calculated to detect changes in ROS while adjusting for any concomitant changes in mitochondrial mass. (**B**) Representative gating of mean fluorescence intensity (MFI) across conditions. (**C**) Quantification of apoptosis rate evaluated by flow cytometry. (**D**) Light microscope images of cultured VSMCs showing cell density after TNFα ± DMF (20×), scale bar (100 µm). All data shown are compiled from three independent experiments (two-way ANOVA with Dunnett’s post hoc against the control group: **** *p* < 0.0001; ** *p* < 0.01; * *p* < 0.05).

Given the observed effects of DMF on cellular respiration and the known reciprocal interaction between ROS and mitochondrial stress/function, we next asked whether DMF increased oxidative stress through ROS generation. To test this, we labeled VSMCs with MitoTracker Red CM-H2XRos fluorescent dye, which accumulates in active polarized mitochondria, is oxidized by ROS into the fluorescent form, and is then retained in mitochondria upon depolarization [28]. VSMCs were co-labeled with MitoTracker Green, which accumulates within mitochondria regardless of the transmembrane potential, and the ratio of red to green was used to detect changes in ROS while adjusting for any concomitant changes in mitochondrial mass. DMF treatment resulted in enhanced mitochondrial ROS levels 24 h after treatment compared with untreated cells (Figure 8A,B). Since mitochondrial ROS production is known to be associated with the induction of apoptosis [29], DMF treatment resulted in significantly increased apoptosis in stimulated VSMCs (Figure 8C,D).

**Figure 9:**
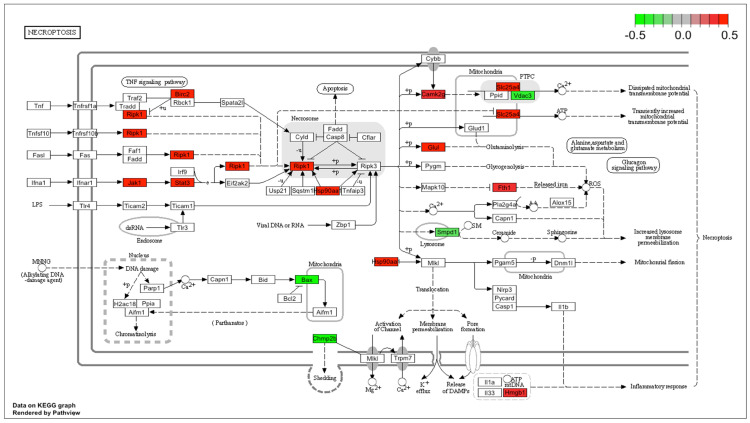
Expression profiles of necroptosis genes visualized on a KEGG pathway diagram using the Pathview package. Red and green indicate genes induced or suppressed by DMF compared to the formed group.

The effect of DMF on cell death response was also confirmed using Pathview analysis from our scRNA-seq data. Fold values of differentially expressed genes (DEGs) were scaled and mapped with appropriate colors for each gene related to the “Necroptosis” pathway, which includes genes that induce ROS generation and open the mitochondrial permeability transition pore. Red and green indicate statistically significant gene up- and down-regulation, respectively. The color grey indicates genes whose expression did not significantly change (Figure 9). Most of the genes displayed in “Necroptosis” were upregulated in VSMCs from the DMF group compared to those from the “formed” group, which is in line with the in vitro results. Taken together, these data indicate DMF affects mitochondrial ROS levels, restricts mitochondrial energy generation and induces caspase-mediated VSMC apoptosis in a concentration-dependent fashion.

### 3.4. DMF Treatment Transiently Depletes Intracellular GSH

**Figure 10:**
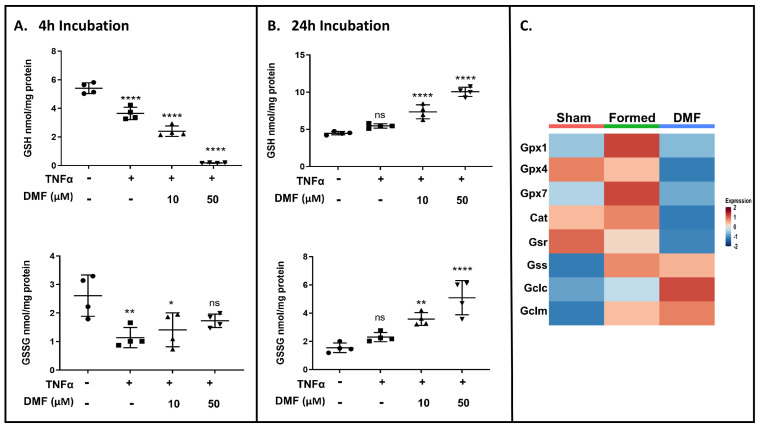
Time course for GSH consumption. Total GSH (**top**) and GSSG (**bottom**) determined at (**A**) 4 h and (**B**) 24 h in VSMCs treated with TNFα ± DMF. (**C**) Heatmap showing relative gene expression levels (columns) of GSH de novo and recycling pathway. All data shown were compiled from three independent experiments (one-way ANOVA with Dunnett’s post hoc against the control group: **** *p* < 0.0001; ** *p* < 0.01, * *p* < 0.05, ns = not significant).

DMF can react as an electrophilic/nucleophilic agent and rapidly deplete GSH circulating levels. We analyzed the total levels of intracellular GSH in cultured VSMCs and found that DMF induced a concentration-dependent transient depletion of intracellular GSH that was recovered after 24 h (Figure 10A,B; top panel). Notably, the increase in GSH levels by DMF occurred with an unexpected and paradoxical increase in intracellular GSSG (Figure 10A,B; bottom panel) and concomitant decrease in the GSH:GSSG ratio (Figure S8A). The increase in total GSH following initial depletion may be a compensatory response.

In vivo, scRNA-seq analysis from VSMC clusters showed that DMF decreased the expression of genes involved in GSH regeneration (Cat, GPx, Gsr) while increasing the expression of the catalytic (Gclc) and modifier (Gclm) subunits of glutamate cysteine ligase (GCL), the rate-limiting enzyme in GSH synthesis (Figure 10A and Figure S8B).

Theoretical predictions of GSH/GSSG ratio support product inhibition of glutathione reductase (Gsr) [30]. Together, these results indicate that DMF Gsr activity disturbs the recycling process, but not de novo synthesis of glutathione (GSH), which would limit the antioxidant capacity of VSMCs under oxidative conditions.

## 4. Discussion

This study aimed to elucidate the impact of DMF exposure on VSMCs in a mouse model of CA using scRNA-seq and to unravel its cell-type-specific response in vitro. The DMF dose chosen overlaps the currently approved human-equivalent doses prescribed for multiple sclerosis (480 mg/day) and psoriasis (720 mg/day). Although DMF and other Nrf2-activator compounds are already used clinically in patients and in ongoing clinical trials, little is known about their impact on healthy and diseased CoW vasculature. Exogenous upregulation of Nrf2 beyond the control of repressive/degradation machinery may have unfavorable consequences, as seen in studies linking upregulation of Nrf2 to cardiac hypertrophy and immune evasion/chemotherapy resistance in cancers [14,31,32]. Previously, our group showed that DMF treatment indeed activated Nrf2 signaling in the cerebral vasculature and significantly inhibited CA progression by modulating VSMC phenotype and function [8]. However, further studies to unveil the complete picture of the effects of DMF in a hypertensive-elastase mouse model of CA were still needed.

We used transcriptomic analysis from subsetted VSMCs and in vitro metabolic assays to help shed light on the mechanisms underlying reduced CA formation with DMF treatment. Our data demonstrated that chronic activation of Nrf2 by DMF (two weeks of 100 mg/kg of DMF per day) (Figure S4) reprograms VSMCs to produce high levels of antioxidants at the expense of mitochondrial bioactivity. As such, DMF treatment resulted in downregulation of nuclear encoded mitochondrial genes and upregulation of pro-apoptotic genes and ROS scavenging genes (Figure 4, Figure 5 and Figure S9). It is well known that Nrf2-based drugs cause irreversible alkylation of cysteine residues on cellular proteins, resulting in side effects [33]. Since DMF is capable of direct alkylation of GSH, it is generally accepted that it also reacts with some critical sulfhydryl (SH) groups of metabolic and regulatory proteins [34]. Notoriously, various SH-targeting alkylating agents negatively affect various mitochondrial functions, including inhibition of ATPase, protein import into mitochondria, bioenergetics and structural integrity [34]. Here, we show that two weeks of DMF treatment after CA induction decreases mitochondrial function (Figure 5) and leads to persistent activation of Nrf2-driven antioxidant gene expression (Figure S4A) that places a burden on cellular bioenergetics.

Multiple studies have demonstrated that DMF has anti-tumor activity in several cancer types by inhibiting proliferation and growth [25,35]. VSMC-derived cells in mouse and human atherosclerosis exhibit multiple tumor cell-like characteristics, including genomic instability, evasion of senescence, hyperproliferation, resistance to cell death, invasiveness, and activation of comprehensive cancer-associated gene regulatory networks [36,37]. In tumorigenic cells, DMF inhibits γ-GCS (the first enzyme of the GSH biosynthesis pathway), leading to less GSH, more ROS and subsequent cell death [35]. Moreover, germline mutations in the fumarate hydratase (FH) gene that predispose to hereditary leiomyomatosis (a benign tumor of smooth muscle origin) and renal cell cancer (HLRCC) cause a buildup of fumarate that leads to the formation of succinic GSH, a covalent conjugate between fumarate and GSH [38]. This chronic succination of GSH, caused by the loss of FH or by exogenous fumarate, culminates in persistent oxidative stress and cellular senescence both in vitro and in vivo [39]. Redox modulation by DMF presents differential effects in malignant vs. nontumorigenic cell lines, and DMF-induced ROS is thought to produce little effect in nontumorigenic cells because they naturally have low levels of oxidizing species [25]. Interestingly, our results suggest otherwise, as DMF rendered VSMCs vulnerable to oxidative stress, leading to mitochondrial dysfunction and apoptosis. Due to the similarities between proliferative VSMCs and cancer cells, and to the fact that hypertension induction contributes to chronic vascular oxidative stress, it is reasonable to assume that, in our mouse model, DMF pushed VSMCs over a critical redox threshold, activating cell death signaling pathways.

Multiple invasive procedures are necessary to generate our CA animal model and produce hypertension that leads to chronic kidney disease (CKD) [40]. Glomerular fibrosis develops in multiple types of CKDs, including diabetic and hypertensive nephropathy [41]; thus, we assessed the effect of DMF on collagen deposition in the glomeruli of the remaining kidney. A significant decrease in glomerular fibrosis was observed in the DMF-treated group compared to the non-treated (Figure S10). This suggests that DMF can halt renal impairment progression. However, other assessments are still needed, but go beyond the scope of this study. It is worth noting that another Nrf2 pathway activator, bardoxolone, was tested in different clinical trials for advanced type 2 diabetic kidney disease and, despite improvement in GFR, there was increased incidence of heart failure among those receiving bardoxolone [42,43]. Moreover, an in vitro study showed that bardoxolone had a negative impact on endothelial bioenergetics and barrier function [44]. Both DMF and bardoxolone are electrophilic chemicals, and these results, taken together, point to detrimental effects of Nrf2 activators on mitochondrial bioenergetics and cellular function in a tissue/cell type-specific manner.

VSMCs can de-differentiate and decrease cellular contractile protein levels in response to environmental cues. Changes in gene expression from the different clusters of VSMCs indicate a spectrum of VSMC phenotypes rather than distinct subsets (Figure 2). The heatmaps highlight the gradual transition between phenotypes in the different treatment groups, with markers of VSMCs related to shape change and transformation (Tagln) and fibroblast maturity (Dcn) being highly expressed in the sham and formed groups, respectively (Figure 2A,D). DMF treatment led to upregulation of Myh11, which encodes smooth muscle myosin heavy chain, and this has been previously shown to result in increased ER stress and autophagy [45]. Protein succination has been shown to be systematically connected with mitochondrial dysfunction, oxidative and ER stress, and apoptosis [46]. Thus, it is possible that DMF restrains the antioxidative capacity of VSMCs to counter pathological intimal thickening, consequently inhibiting CA formation.

Despite its potential damaging effects, we confirmed our previous finding that DMF significantly decreases CA formation in our mouse model. This is not surprising, as excessive VSMC proliferation and migration require increased mitochondrial OXPHOS to meet the increased energy demand for biomass [47,48]; thus, the suppression of mitochondrial oxygen consumption significantly attenuates neointima formation [49,50]. Mechanistically, from scRNA-seq analysis and in vitro assays, we suggest that DMF prevents abnormal VSMC proliferation by disrupting mitochondrial genes and increasing apoptosis. However, while it is important to halt VSMC proliferation to prevent CA formation, it is also imperative to define an optimal intracellular redox environment that can maintain homeostasis and facilitate the regeneration process in response to injury or damage.

Hypertension is prevalent in patients harboring CA and is recognized as a major risk factor for enlargement and rupture [51]. Inducing high blood pressure is an important component of our mouse model; however, this makes sorting out therapeutic mechanisms more challenging because multiple organs are affected (kidney and brain vasculature). Here, we found that two weeks of DMF treatment disturbs the transcription of recycling process genes, but not de novo synthesis of glutathione (GSH), limiting the antioxidant capacity of cerebral VSMCs in the higher ROS environment that is created when CA is induced. Consequently, this elicits changes in VSMC mitochondrial gene expression and leads to the loss of mitochondrial activity. The GSH/GSSG recycling enzymes play a central role in oxidative stress management and, therefore, suppressing them may cause a fatal increase in ROS and cell death [52]. One of the mechanisms associated with GSH recycling disruption involves direct inhibition of the glutathione peroxidase (GPx) subunit Gpx4. Of note, fumarate accumulation may lead to succination of Gpx4 at cysteine 93, which significantly reduces its enzymatic activity [53,54]. In this study, DMF treatment led to the downregulation of several genes belonging to the GPx family, including Gpx4 (Figure S8B). Gpx4 inhibition contributes to lipoxygenase activation and calcium influx, which induces mitochondrial permeability pore (MPTP) opening and necroptosis via induction of ROS accumulation [55]. Accordingly, our Pathview analysis revealed elevated expression of genes related to necroptosis when animals were treated with DMF (Figure 9), indicating that DMF-induced GSH disruption contributes to DMF-induced cell necroptosis. A similar effect has been described in different cell types where elevated mitochondrial ROS production and the resulting oxidative stress were found to be directly involved in DMF-mediated modulation of key immune functions of T cells and macrophages [27,56]. Importantly, human patients starting DMF therapy display an increased level of non-enzymatically produced isoprostanes, which strongly supports a net increase in their oxidative state, at least in the peripheral blood [27].

Ultimately, the benefit of a drug to a patient population weighs efficacy against the unwanted side effect profile. Hence, timing of exposure to DMF and dosage can be critical for the outcome. One limitation of this study is that only one regimen of DMF was tested. Additionally, DMF was given systemically (oral gavage). Thus, it will be important to evaluate different regimen strategies and targeted delivery of DMF using lipid-based nanocarriers [57] to decide if it can be used as effective therapy for CA prevention. Limitations and potential biases should also be considered when analyzing and interpreting scRNA-seq datasets. The data presented here relied on a single-cell suspension representative of CoW tissue exposed to tissue disruption, and this process can cause variations in transcript levels that might not reflect differences in protein levels or cellular functions. Moreover, the lack of depth of scRNA-seq also makes it difficult to investigate low-expression genes, which are often transcription factors [6].

## 5. Conclusions

Taken together, our data support the concept that the DMF-mediated antiproliferative effect on VSMCs is linked to disturbed antioxidative functions resulting in altered mitochondrial metabolism. Therefore, before severe adverse effects emerge, it would be clinically relevant to develop indices or biomarkers linked to this disturbed antioxidative function to monitor patients undergoing DMF treatment. An optimal treatment for CA prevention should maintain a healthy balance between ROS and antioxidants, as both extremes, oxidative and antioxidative stress, are damaging. Since DMF can both induce and inhibit oxidative stress, one alternative for individualized optimization of therapeutic responses might be dose adaptation according to the GSH recycling depletion status. Furthermore, because DMF modifies active cysteines in proteins affecting various cellular pathways, resulting in side-effects, our future studies will evaluate supplementation with L-cysteine and N-acetyl-L-cysteine (NAC) to alleviate the oxidative stress damage caused by DMF [56,58] in the context of CA formation. A limitation of the in vivo findings is that they relied on scRNA-seq, which does not provide insight into downstream functional consequences. Future investigations will integrate scRNA-seq analysis with other single cell -omic data to link these networks together and provide additional value in decoding complex relationships of CA formation reduction and DMF treatment.

## Data Availability

The scRNAseq data generated in this study can be downloaded from the Gene Expression Omnibus (GEO) database using the accession codes GSE193533 and GSE268079.

## Supplementary Materials

The following supporting information can be downloaded at: https://www.mdpi.com/article/10.3390/antiox13070773/s1, Figure S1: Klf4 gene expression level in subpopulations of VSMC; Figure S2: Representative brain sections demonstrating expression of αSMA (green) and nuclear stain DAPI (blue). Figure S3: Heatmap from genes identified using pseudotime analysis in VSMCs across conditions. Figure S4: Impact of DMF on the expression levels of Nrf2-related genes. Figure S5: Mitochondrial DNA (mtDNA)-encoded genes involved with oxidative phosphorylation in VSMCs subpopulations across conditions. Figure S6: Seahorse XF real-time ATP rate analysis of VSMCs treated with ML385. Figure S7: Seahorse XF real-time glycolysis stress test and extracellular lactate levels of VSMCs. Figure S8: Rate-limiting enzymes in GSH regeneration and recycling in VSMCs subpopulations across conditions. Figure S9: Expression profiles of fluid sheer stress and atherosclerosis genes visualized on an KEGG pathway diagram using the Pathview package. Figure S10: Masson’s trichome-stained images of kidney showing glomerular fibrosis. Table S1: Top100 DEGs in VSMCs [59,60,61,62,63].

## Abbreviations

ATP, adenosine triphosphate; CMC, carboxymethylcellulose; CA, cerebral aneurysm; CKD, chronic kidney disease; CoW, circle of Willis; DMF, dimethyl fumarate; ER, endoplasmic reticulum; ECAR, extracellular acidification rate; FH, fumarate hydratase; GSH, glutathione; MTD, maximum tolerable dose; OXPHOS, oxidative phosphorylation; ROS, reactive oxygen species; scRNA-seq, single-cell RNA sequencing; VSMC, vascular smooth muscle cell.
