# Supplementary material for: Dimethyl Fumarate Mediates Sustained Vascular Smooth Muscle Cell Remodeling in a Mouse Model of Cerebral Aneurysm
Source: Antioxidants (Basel). 2024 Jun 27;13(7):773. doi: 10.3390/antiox13070773 (PMC11274241; doi:10.3390/antiox13070773)
Supplement: Supplementary file 1 [file antioxidants-13-00773-s001.zip › Supplementary Materials Figures.pdf]

Dimethyl Fumarate Mediates Sustained Vascular Smooth Muscle Cell Remodeling in a Mouse Model of Cerebral Aneurysm

Alejandra N. Martinez [†], PhD [1], Giovane G. Tortelote, PhD [2], Crissey L. Pascale, MA [1], Uduak-Obong I. Ekanem, MS [1], Ana Paula de O. Leite, PhD [3], Isabella G. McCormack, MS [1] and Aaron S. Dumont, MD [1]

[1] Department of Neurosurgery and The Tulane Center for Clinical Neurosciences, Tulane University School of Medicine 1430 Tulane Avenue New Orleans, LA 70012, USA
[2] Department of Pediatrics, Tulane University School of Medicine, New Orleans, LA 70112, USA
[3] Department of Pharmacology and The Tulane Center for Sex-Based Biology and Medicine, Tulane University School of Medicine, New Orleans, LA 70112, USA
[†] Correspondence: amarti8@tulane.edu, 504-988-7608.
Address of corresponding author: Department of Neurosurgery, Tulane Center for Clinical Neurosciences, Tulane University School of Medicine 1430 Tulane Avenue (Room M731), New Orleans, LA 70012

Supplementary Methods

Mouse model of elastase-induced CA

Female mice were not used because previous work in our lab has shown no detectable difference in aneurysm outcome between sexes at this age. The number of animals was chosen to ensure an adequate number of mice (n=5) would survive to completion in each experimental outcome (formed and ruptured). There is a 35% mortality rate associated with this model due to the surgery itself, incomplete recovery post-surgery and ruptured aneurysms. Only mice that survived until experimental completion at the two week time point were used in the study and the tissue from any extra mice that survived until the end of the study was used in other lab experiments. For all survival surgeries, anesthesia was established using ketamine and xylazine at 100 & 10mg/kg, respectively. To induce aneurysm formation in mice we used a well-established model that involves pharmacological hypertension and stereotactic injection of elastase into the basal cistern [17]. Briefly, the mice underwent a unilateral nephrectomy followed by implantation of a deoxycorticosterone acetate (DOCA) pellet (Innovative Research of America; M-121 50mg, 21day release) in the subcutaneous tissue of the interscapular region one week

later. Also, at that time, the mice were given 1% NaCl in their drinking water and elastase (Sigma Aldrich; E7885) was injected into the basal cistern.

To perform the stereotactic injection of elastase into the basal cistern, the tip of a 26 g needle was positioned using the following coordinates on a stereotaxic frame: 1.3mm lateral to the midline, 2.6mm posterior from bregma and 6 mm ventral to the skull, and elastase (17.5mU in 2.5μL sterile PBS) was injected at a rate of 0.2μL/min. Sham animals were not made hypertensive and had PBS injected instead of elastase. All mice underwent daily health checks thereafter. At the conclusion of the experiment, the brains were harvested and the CoW dissected under a microscope. A formed aneurysm was designated as any outward bulging of a vessel that was at least 150% larger than the parent vessel.

Single-cell RNA-sequencing data analysis

For 10x single cell RNAseq assay, ~5000 live cells per sample were targeted by using 10x Single Cell 3' RNAseq technology provided by 10x Genomics (10X Genomics Inc, CA). Briefly, viable single cell suspensions were partitioned into nanoliter-scale Gel Beads-In-EMulsion (GEMs). Full-length barcoded cDNAs were then generated and amplified by PCR to obtain sufficient mass for library construction. Following enzymatic fragmentation, end-repair, A-tailing, and adaptor ligation, single cell 3' libraries comprising standard Illumina P5 and P7 paired-end constructs were generated. Library quality controls were performed by using Agilent High Sensitive DNA kit with Agilent 2100 Bioanalyzer and quantified by Qubit 2.0 fluorometer. Pooled libraries at a final concentration of 750pM were sequenced with paired-end single index configuration by Illumina NextSeq 2000. Cell Ranger version 6.0.1 (10x Genomics) was used to map raw data to references of gex-mm10-2020-A. All subsequent analysis was performed using R programing language. Assessment of sequence quality control, normalization, clustering and low dimension projections calculations were performed in R. Seurat (v4.02) [59]. Cells with no counts or less than 300 genes detected, or more than 15% of mitochondrial genes were removed. Cluster identity was determined using 4000 highly variable genes, and the first 20 principal components. We obtained lists of DE genes for each cluster using the Seurat FindAllMarkers function and upregulated genes in each cluster were used to classify the different cell types. The differential gene expression between treatments was performed with Seurat FindAllMarkers function with the default arguments settings. To further understand gene biological functions and associated pathways, we performed the gene ontology (GO) and KEGG pathway enrichment analysis with the Goanna

(from limma), and additional unsupervised and supervised ontology with ShinyGo [60]. For KEGG pathways data integration and visualization we used the R package Pathview [61]. The DEG list between treatments was used as input as described in the figure legend. Pseudotime analysis was performed using Slingshot [62].

Kidney histological evaluation and quantification

The remaining kidneys from each experimental group (n = 8 per group) were collected at sacrifice, immersion-fixed with 4% paraformaldehyde buffer (pH 7.4) for 24 h and then OCT embedded. Serial sections of 5–7 µm thickness were cut, and subjected to Masson's trichrome staining technique that allows the visualization of collagen fibers, with fibers appearing red, nuclei black, and collagen blue. From each kidney, a total of 5 random glomeruli were captured at 40-fold magnification using under identical conditions at the same magnification using a light microscope (Olympus CKX41). The images were evaluated by using Image-Pro Plus 4.1 software (Media Cybernetics, Silver Spring, USA). The positive area was calculated as a % of the total area as previously described [63].

Immunocytochemistry and confocal microscopy

A subset (n = 4) of mice had the entire brain harvested for immunohistochemistry. They were placed in 4% paraformaldehyde overnight (o/n), followed by 15% sucrose for 24 h, and, finally, 30% sucrose for 24 h. They were then placed in OCT media and frozen using liquid nitrogen. Coronal sections were obtained at a thickness of 16 µm. Representative brain sections were blocked for 1 h using blocking buffer (PBS containing 1% BSA, 0.1% cold fish skin gelatin, 0.5% Triton X-100 and 0.05% sodium azide) and, subsequently, incubated with the following primary antibodies: anti-actin α-smooth muscle (Sigma Aldrich A2547; 1:200 dilution) for 2 h at RT. Alexa Fluor 488 (1:600; Life Technologies) was applied for 1 h, followed by nuclear labeling with DAPI (1:300; Life Technologies) for 5 min. ProLong Gold mounting media (Life Technologies) was used to prevent photobleaching during imaging. Stained slides were imaged using the Nikon eclipse Ti2 confocal microscope. The software Image J was used for fluorescence intensity quantification analysis that was normalized by cell count using DAPI.

Supplemental figure legends

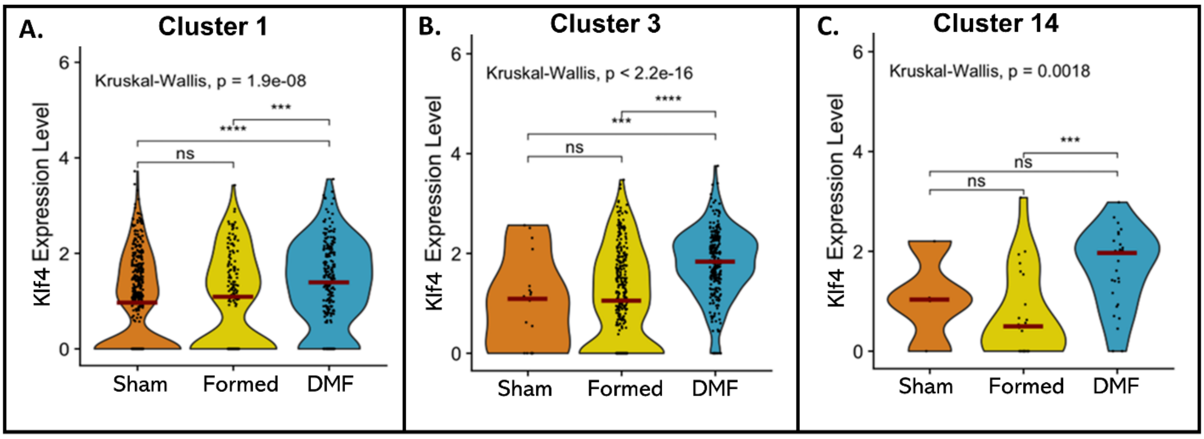

Figure S1. Klf4 gene expression level in subpopulations of VSMC. Violin plot of Klf4 gene expression across VSMC conditions on the single cell level. **(A)** Cluster 1, **(B)** Cluster 3 and **(C)** Cluster 14. Each dot represents a single cell. Kruskal-Wallis test was used to test overall significance.

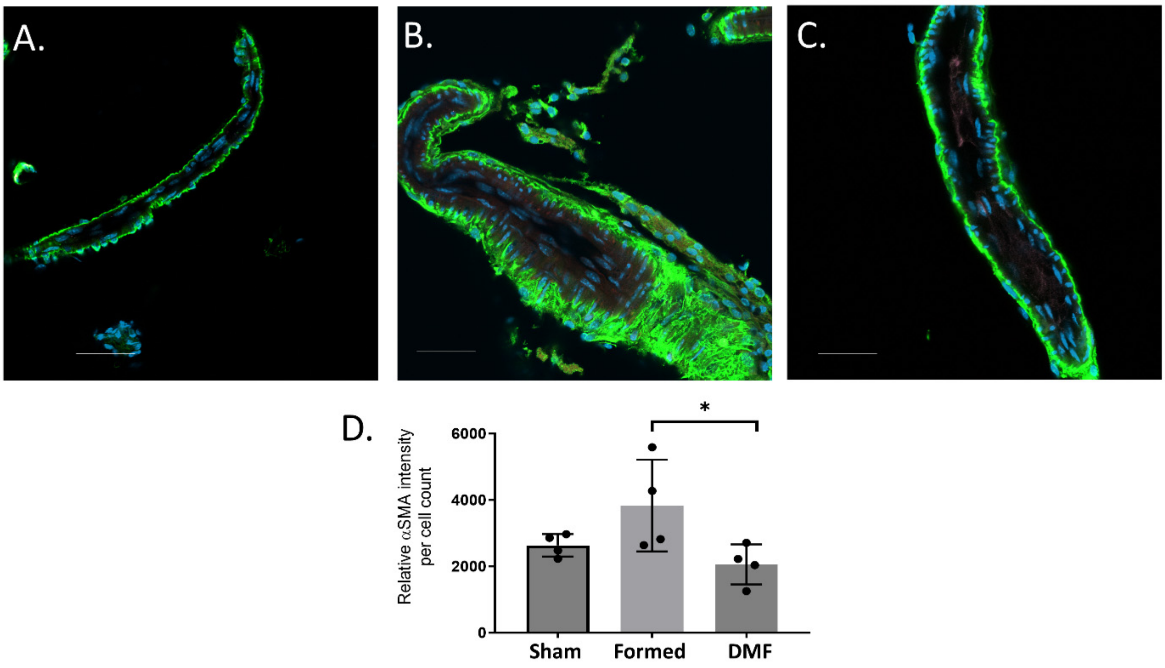

Figure S2. Representative brain sections demonstrating expression of αSMA (green) and nuclear stain DAPI (blue) in **(A)** sham operated animal, **(B)** CA induced animal (Formed) and **(C)** DMF treated animal. Confocal images taken using Nikon eclipse Ti2 microscope and 60X magnification (scale bar, 10µm). **(D)** Quantification of SMA-α deposition normalized by cell count using DAPI (n = 4 brains per group). Statistical significance as demonstrated by one-way ANOVA with Tukey's post hoc; $*p < 0.05$.

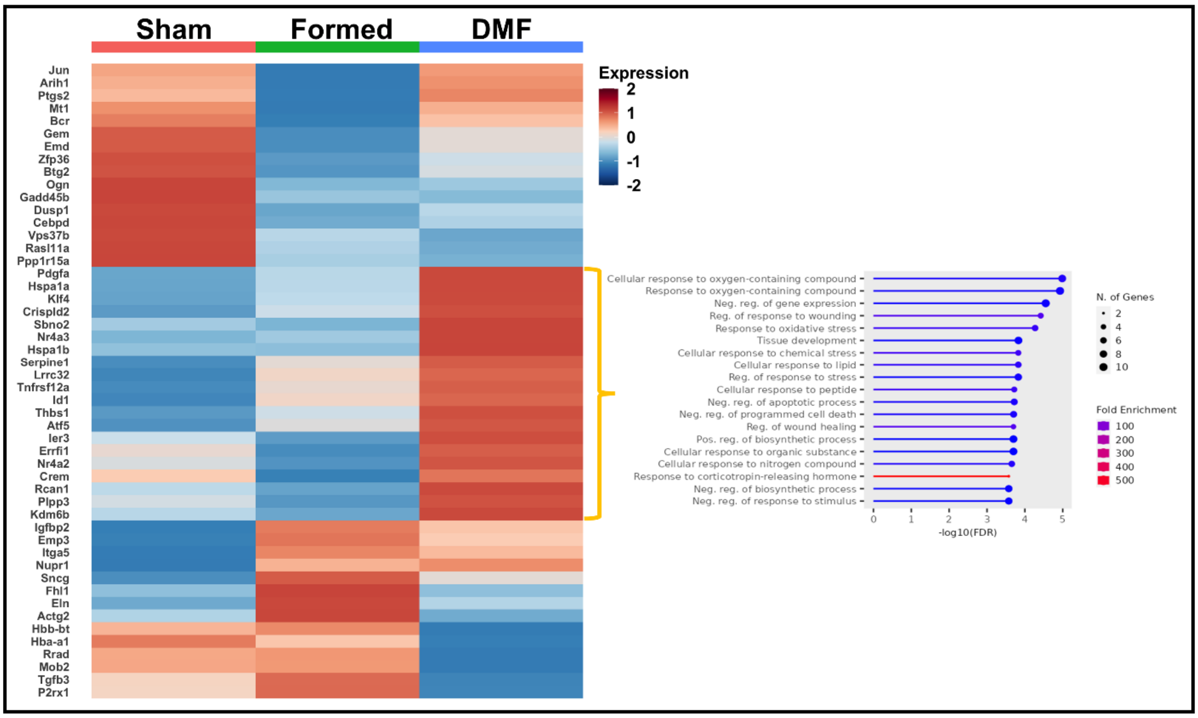

Figure S3. Heatmap from genes identified using pseudotime analysis in VSMCs across conditions. Bracket encompasses the gene ontology enrichment analysis of biological processes from differentially expressed genes in the DMF group using ShinnyGO 0.77.

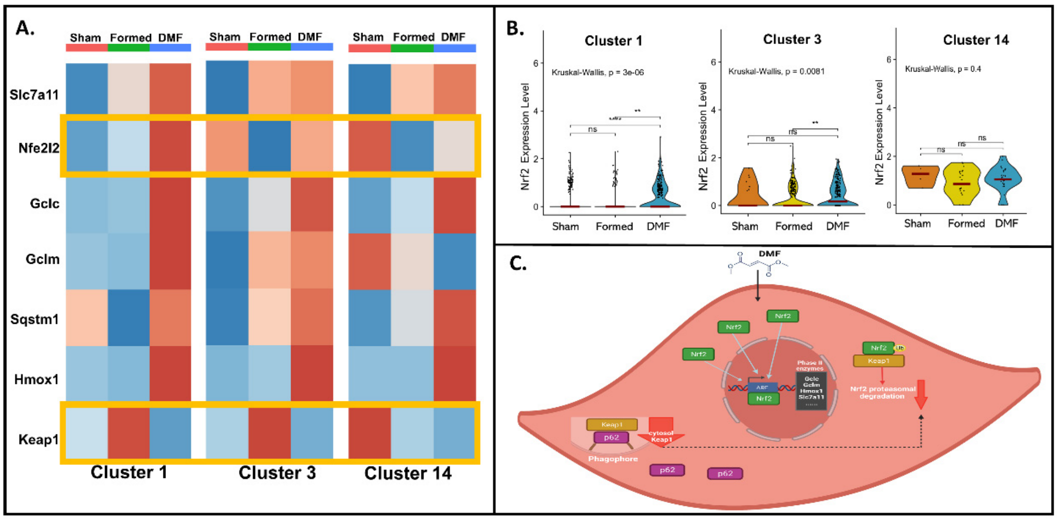

Figure S4. Impact of DMF on the expression levels of Nrf2-related genes. **(A)** Heatmap showing the expression profile of Nrf2-related genes in subpopulations of VSMCs across conditions. Yellow rectangles highlight Nrf2 upregulation and Keap1 downregulation. **(B)** Violin plot of Nrf2

gene expression across VSMC conditions on the single cell level. Each dot represents a single cell. Kruskal-Wallis test was used to test overall significance. **(C)** Scheme depicting the activation state of Nrf2 by DMF in VSMCs matching to the transcriptional profile. Two weeks of DMF treatment leads to activation of the Nrf2 through succination of the cysteine residues of Keap1, promoting its degradation via proteasome or via sequestosome (p62). The latter induces the nuclear translocation of Nrf2 to interact with and bind to antioxidant response elements (AREs), triggering the expression phase II enzymes. The figure was created using BioRender.

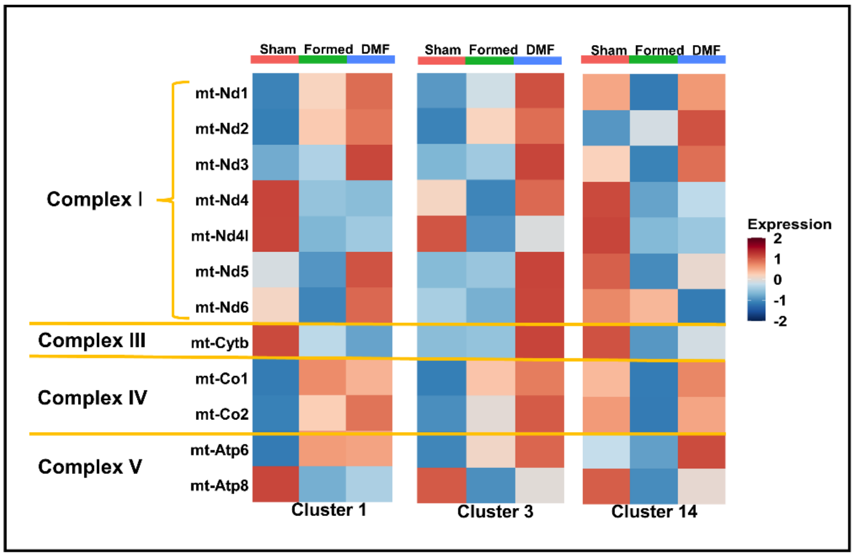

Figure S5. Mitochondrial DNA (mtDNA)-encoded genes involved with oxidative phosphorylation in VSMCs subpopulations across conditions. Heatmaps showing changes in transcript levels for specific mitochondrial complex across each individual VSMC cluster and treatment group.

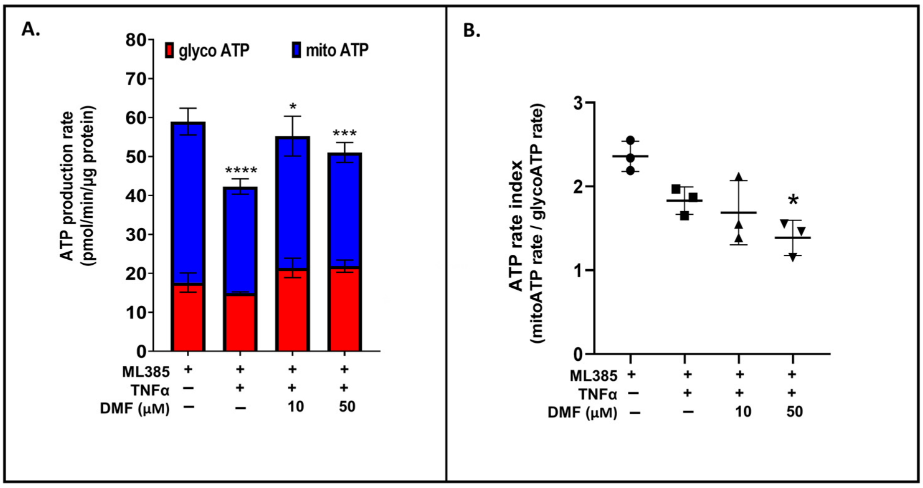

Figure S6. Seahorse XF real-time ATP rate analysis of VSMCs treated with ML385. **(A)** Mitochondrial and glycolytic ATP production rates in VSMCs treated with ML385 and TNFα ± DMF. **(B)** XF ATP rate index calculated from data in panel A. All Seahorse data shown are compiled from two independent experiments using four technical replicates per experiment

per condition and normalized by protein content (two-way ANOVA with Dunnett's post hoc against the control group: ****p < 0.0001; **p < 0.01; *p < 0.05.

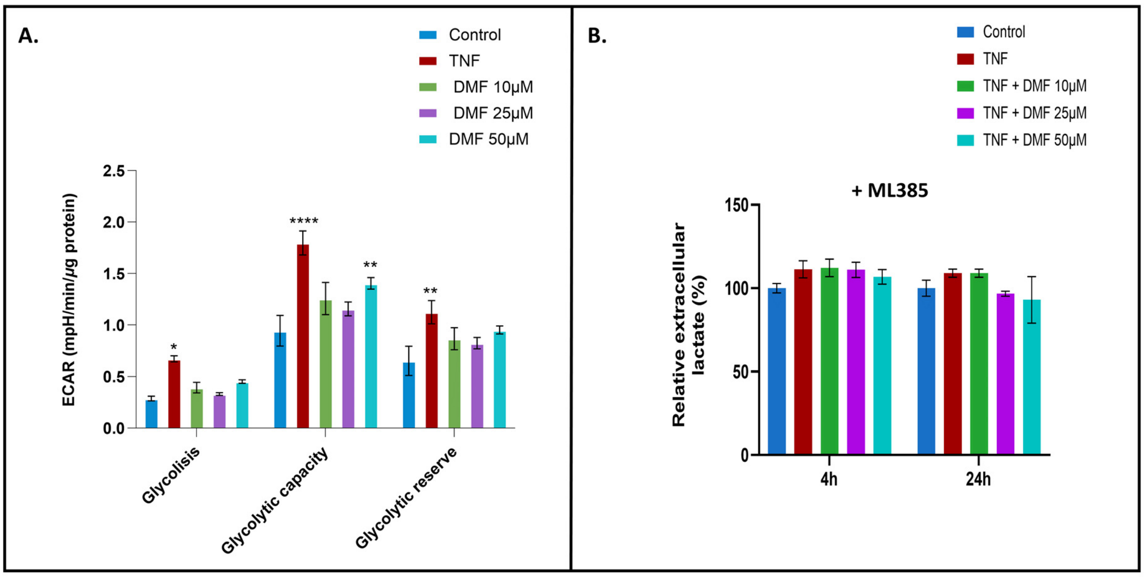

Figure S7. Seahorse XF real-time glycolysis stress test and extracellular lactate levels of VSMCs. **(A)** Glycolysis and Glycolytic bioenergetic parameters: basal glycolysis, glycolytic capacity and glycolytic reserve measured in VSMCs treated with TNFα or DMF alone. **(B)** Extracellular lactate levels in VSMC supernatant treated with ML385. Relative levels of lactate in the culture supernatant of VSMCs treated for 4 and 24 hours withML385 and TNFα ± DMF. All data shown are compiled from two independent experiments using three technical replicates per experiment per condition and normalized by protein content (two-way ANOVA with Dunnett's post hoc against the control group showed no statistical significance between the groups compared).

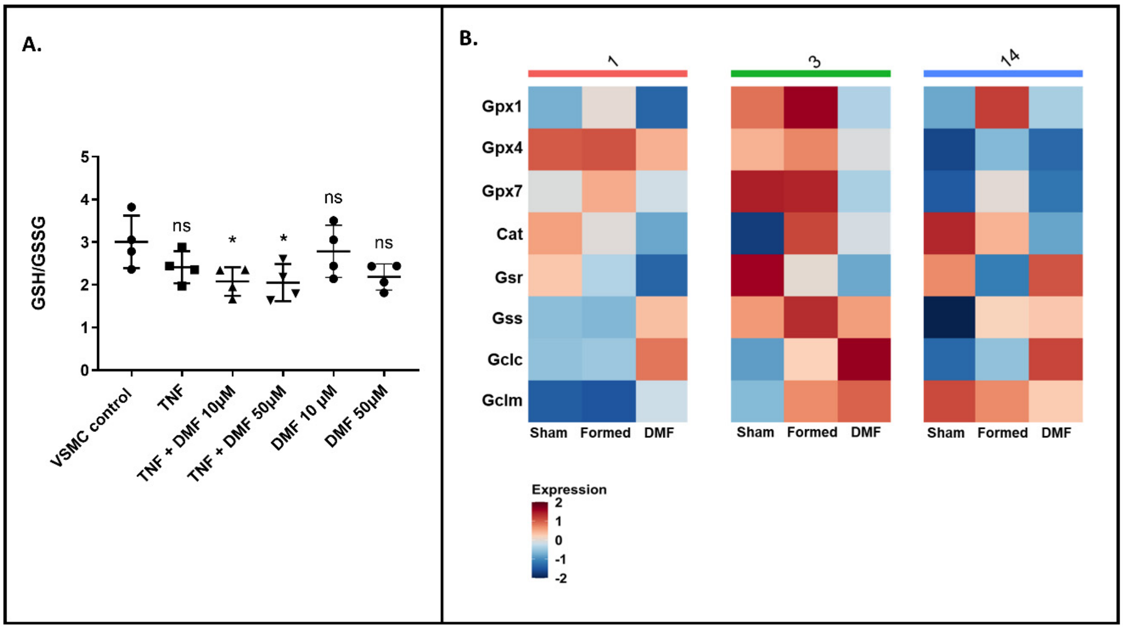

Figure S8. Rate-limiting enzymes in GSH regeneration and recycling in VSMCs subpopulations across conditions. **(A)** GSH/GSSG ratio. **(B)** Heatmap showing relative gene expression levels (columns) of GSH *de novo* and recycling pathway per cluster and treatment group.

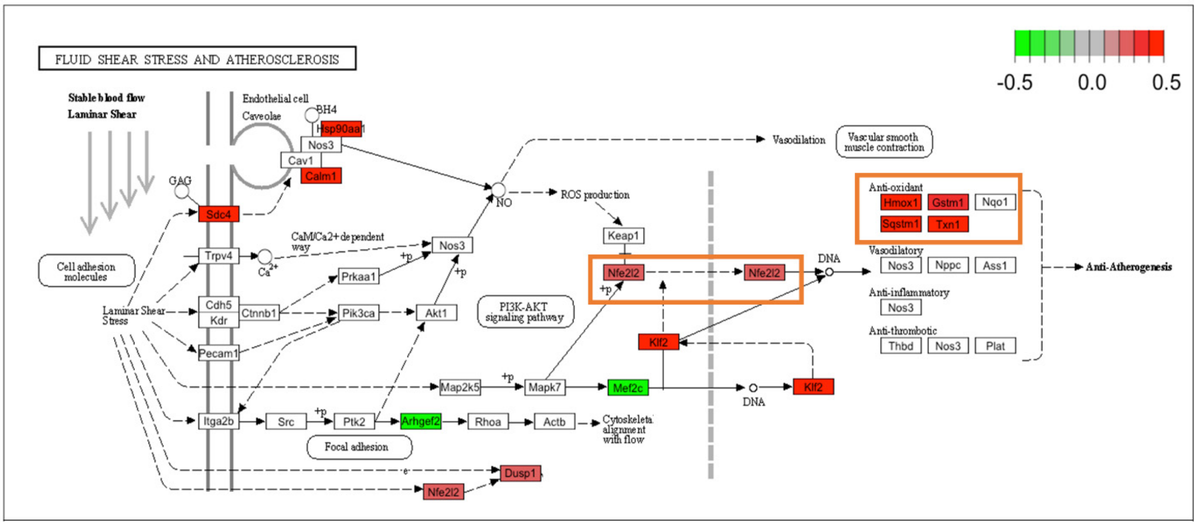

Figure S9. Expression profiles of fluid sheer stress and atherosclerosis genes visualized on an KEGG pathway diagram using the Pathview package. Red and green indicate genes induced or suppressed by DMF compared to the formed group.

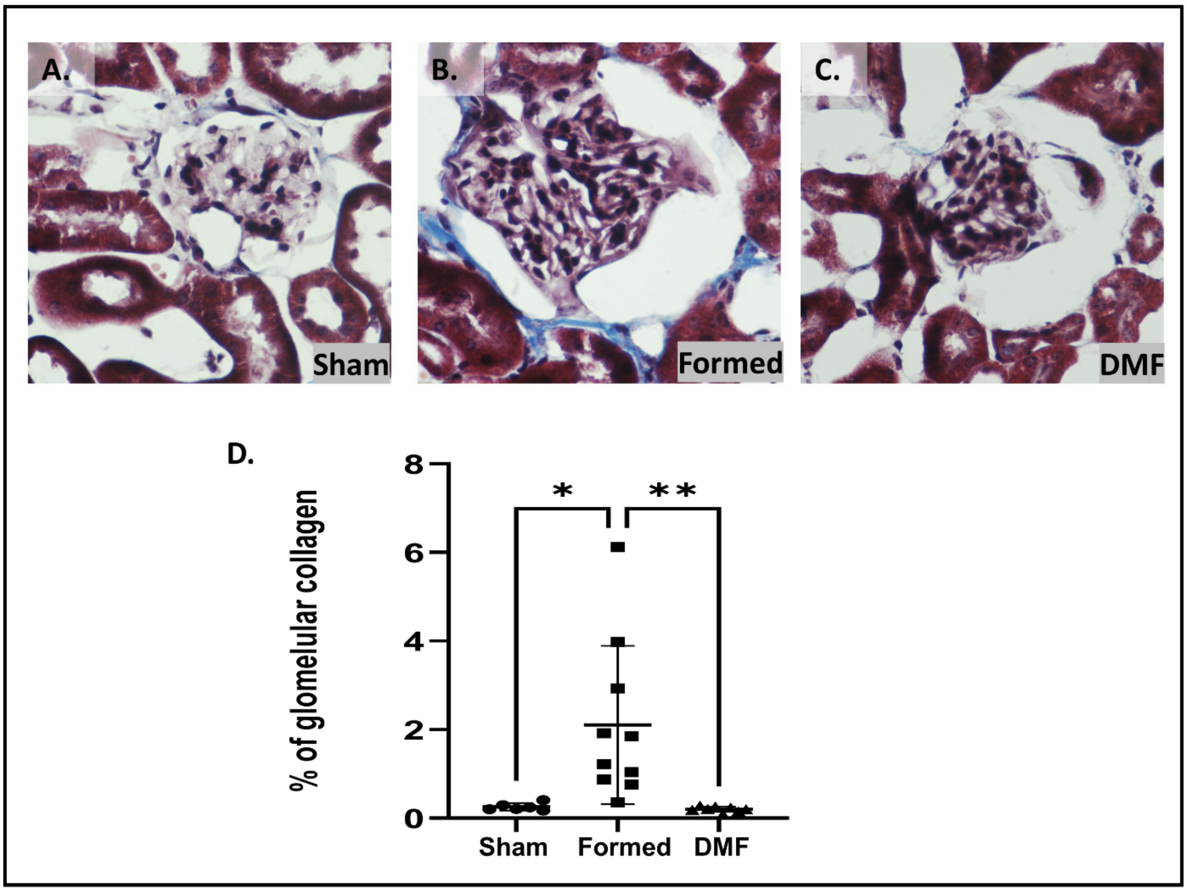

Figure S10. Masson's trichome-stained images of kidney showing glomerular fibrosis under 40X magnification. Representative kidney sections from **(A)** Sham operated animal, **(B)** CA induced animal (Formed) and (**C)**) DMF treated animal. **(D)** Quantification of glomerular collagen deposition. Statistical significance as demonstrated by one-way ANOVA with Tukey's post hoc; $^{**}p < 0.01$; $^{*}p < 0.05$.
